# Comparison of the performance of a clinical classification tree versus clinical gestalt in predicting sepsis with extended-spectrum beta-lactamase–producing gram-negative rods

**DOI:** 10.1017/ash.2021.253

**Published:** 2022-03-07

**Authors:** Hayden L. Smith, Mohamed A. Elfeki, Dana Lowry, Rebecca Sabates, Molly Ropte, Katherine R. Sittig, Stephanie Finch, Kristina Braun, William Pruett, Aaron Wasson, Kenna Fischman, Jennifer Peterson, Rossana Rosa

**Affiliations:** 1 Iowa Methodist Medical Center, UnityPoint Health-Des Moines, Des Moines, Iowa; 2 Internal Medicine Department, University of Iowa, Des Moines, Iowa; 3 Des Moines University College of Osteopathic Medicine, Des Moines, Iowa; 4 Infectious Diseases Service, UnityPoint Health–Des Moines, Des Moines, Iowa

## Abstract

A clinical decision tree was developed using point-of-care characteristics to identify patients with culture-proven sepsis due to extended-spectrum β-lactamase–producing *Enterobacterales* (ESBL-PE). We compared its performance with the clinical gestalt of emergency department (ED) clinicians and hospital-based clinicians. The developed tree outperformed ED-based clinicians but was comparable to inpatient-based clinicians.

Infections with extended-spectrum β-lactamase–producing *Enterobacterales* (ESBL-PE) are associated with a higher mortality risk compared to infections by susceptible organisms,^1^ due partly to inadequate empirical antimicrobial therapy.^2^ Carbapenems are the preferred agents for the treatment of bloodstream infections due to ESBL-PE,^3^ but their widespread use can lead to acquisition of carbapenem-resistant organisms.^4^

Clinical decision trees (hereafter referred to as decision trees) have been proposed as a tool to determine a patient’s probability of infection with an ESBL-PE to better optimize empirical antimicrobial therapy.^5,6^ However, the applicability of existing decision trees might be limited by baseline antimicrobial resistance rates and specific demographic characteristics of the cohorts used to build them. We built a decision tree to identify patients presenting with sepsis due to ESBL-PE in a setting with low prevalence of resistance and compared it to clinicians’ ability to correctly choose empiric antimicrobial therapy.

## Methods

We conducted a retrospective review of medical records from 3 hospitals within a health system in Des Moines, Iowa, from July 2015 to December 2019. We first identified adult patients that met the Sepsis-2 criteria for sepsis, severe sepsis, or septic shock at the time of presentation to the emergency department (ED) and had a blood culture obtained as part of their evaluation.^7^ These definitions comprised the criteria applied at our hospitals during the study period. The final cohort included only individuals with culture-proven infection defined as growth of Enterobacterales in at least 1 blood culture. We included the first episode of bacteremia per patient. Patients with growth from only nonsterile sites (eg, urine and sputum) were excluded due to difficulties in differentiating colonization versus infection.

The study outcome was bloodstream infection with an ESBL-PE (Supplementary Document 1). Based on previous studies, the point-of-care variables of interest were age, documented history of ESBL-PE colonization or infection in prior 2 years, documented antibiotic use in prior 6 months, care facility residence, and central line present on admission.^5^ Empirical administration of a carbapenem was used as a surrogate indicator of a clinician’s level of suspicion (ie, clinical gestalt) for infection with an ESBL-PE. Because empiric antibiotic selection choices between ED and hospital-based clinicians often varies, we decided to evaluate these 2 groups separately.

We estimated descriptive statistics using a classification tree (r package: rpart version 4.1-13 software, R Foundation for Statistical Computing, Vienna, Austria) was fit with ESBL-PE status as the dependent variable and point-of-care information was fit as the independent variable. ESBL-PE status was considered a potential rare event in the patient population. Steps were taken to control threats related to analyzing an imbalanced outcome (Supplementary Document 2).

The final pruned tree was fit to the full data set, and ESBL-PE screening metrics included the following: sensitivity, specificity, positive predictive value, negative predictive value, positive likelihood ratio, negative likelihood ratio, and accuracy. The area under the receiver operating characteristic curve (AUROCC) for the final tree was compared with both the AUROCC for patients prescribed a carbapenem by the ED or by admitting hospital-based clinician. Further details of the tree-building process are presented in Supplementary Document 2. The study protocol was reviewed and approved by the local institutional review board.

## Results

The study sample included 621 patients, with 56 (9%) were positive for an ESBL-PE infection. Patient characteristics stratified by ESBL-PE status are presented in Table 1 and Supplementary Figure 1. Of the study patients, 28 (4%) had a history of ESBL-PE infection or colonization in the previous 2 years, 97 (16%) came from a care facility, and 28 (5%) had a central line present on admission.

**Table 1.** Emergency Department Point-of-Care Information Available in Patients Presenting With Sepsis, Severe Sepsis, or Septic Shock (N = 621)

Variable	ESBL-PE Positive (n = 56), No. (%)^a^	ESBL-PE Negative (n = 565), No. (%)^a^
Sex, female^b^	32 (57)	339 (60)
Age, median y (SD)	72 (13)	66 (18)
Documented history of ESBL in last 2 y	21 (38)	7 (1)
Documented history of antibiotic use in prior 6 mo	35 (63)	243 (43)
Care facility residence	24 (43)	73 (13)
Central line present on admission	1 (2)	27 (5)
Received carbapenem from emergency department^b^	27 (48)	48 (8)
Received carbapenem from hospital-based service^b^	4 (7)	23 (4)
**Organism** ^**c**^		
*Escherichia coli*	51 (91)	339 (60)
*Klebsiella pneumoniae*	1 (2)	113 (20)
*Klebsiella oxytoca*	1 (2)	18 (3)
*Enterobacter* spp	1 (2)	29 (5)
*Citrobacter* spp	0 (0)	6 (1)
*Morganella morganii*	0 (0)	4 (1)
*Serratia marcescens*	0 (0)	7 (1)
*Proteus* spp	0 (0)	29 (5)
*Polymicrobial*	2 (4)	20 (4)

Note. ESBL-PE, extended-spectrum β-lactamase–producing *Enterobacterales*; SD, standard deviation.

a Units unless otherwise specified.

b Not used in tree as point-of-care information.

c Not point-of-care information.

**Fig. 1.**
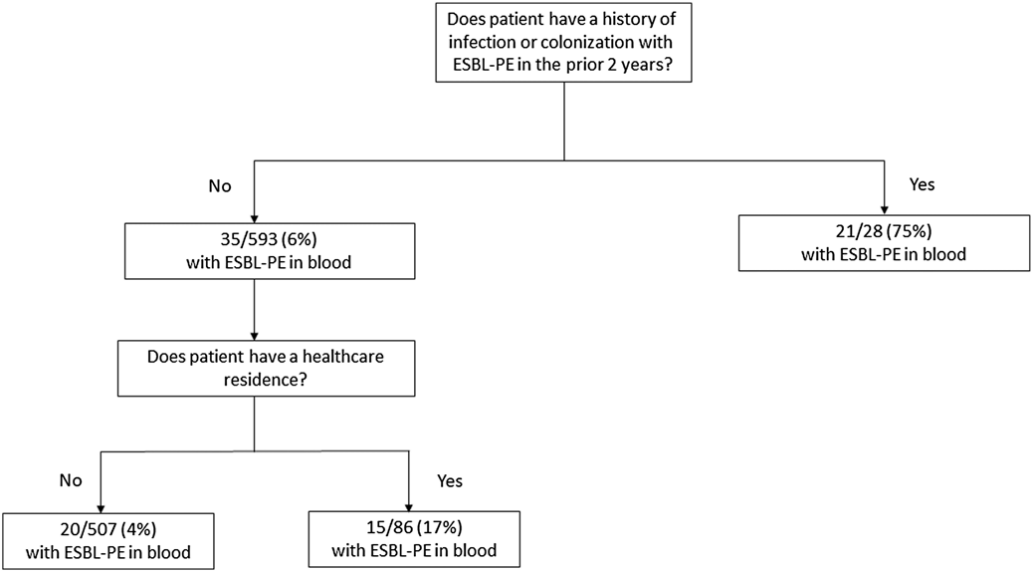
Classification tree fit based on emergency department point-of-care information available in patients presenting with sepsis, severe sepsis, or septic shock (N = 621). Overall, 56 patients were ultimately determined to have culture-proven sepsis with extended-spectrum β-lactamase–producing *Enterobacterales* (ESBL-PE) in blood, representing 9% of the sample.

The selected decision tree for ESBL-PE status included 2 splits. The first split was between patients with a documented history of ESBL-PE and patients without an ESBL-PE history. These groups were then split based on whether they lived in a care facility (Fig. 1 and Supplementary Fig. 2). Patients with a history of ESBL-PE had a 71% (95% CI, 53%–90%) higher rate of ESBL-PE than patients without a history of ESBL-PE and non–care-facility residence. Patients without a history of ESBL-PE, but with care-facility residence, had a 14% (95% CI, 4%–23%) higher rate of ESBL-PE than patients without an ESBL-PE history and a non–care-facility residence. The sensitivity and specificity of the tree were 0.64 (95% CI, 0.50–0.77) and 0.86 (95% CI, 0.83–0.89), respectively. The positive predictive value and negative predictive value were 0.32 (95% CI, 0.23–0.41) and 0.96 (95% CI, 0.94–0.98), respectively. The positive likelihood ratio was 4.66 (95% CI, 3.51–6.18) and the negative likelihood ratio was 0.41 (95% CI, 0.29–0.59).

The tree had an accuracy of 0.84 (95% CI, 0.81–0.87) and its calibration curve is presented in Supplementary Figure 4. The AUROCC for the tree was 0.77 (95% CI, 0.71–0.84), and the AUROCC for whether the patient was prescribed a carbapenem empirically by the ED clinician was 0.52 (95% CI, 0.48–0.55) (Supplementary Fig. 3). The difference between these 2 values was 0.26 (95% CI, 0.19–0.33; S value, 40.9). The AUROCC value for whether a patient was empirically prescribed a carbapenem by the admitting service was 0.70 (95% CI, 0.63–0.77). Hypothetically, using the tree in addition to the hospital-based clinicians’ recorded practice patterns would result in a combined AUROCC of 0.83. Sensitivity analyses revealed fitting a tree based on ESBL-PE balancing via equal prior probabilities would result in an identical tree as the one presented in this paper.

## Discussion

In the presented study, the combination of a history for ESBL-PE colonization or infection and residence in a care facility had a NPV in the upper ninety percentiles in a sample with a low prevalence of ESBL. Although both variables have been independently associated with an increased risk of infection with ESBL-PE,^5,6,8,9^ we showed that application of a decision tree making use of these 2 point-of-care variables can help better identify patients and potentially guide therapeutic decisions. Furthermore, the decision-tree accuracy to identify individuals who would benefit from empiric carbapenem therapy was higher than that of ED clinicians’ gestalt alone. Also, the decision-tree accuracy was comparable to that of hospital-based clinicians’ gestalt but without full overlap. This latter result could be indicative of the impact of the local antibiotic stewardship program (ASP), which frequently engages with hospital-based clinicians using prospective review and feedback. It has been recognized that ASPs should work more closely and collaboratively with ED clinicians to improve antimicrobial use.^10^

This study had several limitations. The sample was taken from a medium-sized city with a relatively homogeneous population and low prevalence of ESBL-PE, which constrained the number of variables that could be examined in the tree-building process and limits the generalizability of our results. We created a classification tree and acquired its estimates based on a single data sample, which may result in an overly optimistic generalizability. This concern was partially lessened by cross validation in the tree-building process. Furthermore, stronger evidence in favor of carbapenems as the preferred agent in the treatment of ESBL-PE was published toward the end of the study period, which could have changed prescribing practices.^3^

A decision tree derived from variables available at point-of-care can help assess the need for empiric carbapenem use among patients with culture-proven sepsis due to *Enterobacterales*. Antibiotic stewardship programs should consider engaging ED clinicians when deploying strategies for improving antimicrobial use.

## Supplementary material

For supplementary material accompanying this paper visit https://doi.org/10.1017/ash.2021.253. click here to view supplementary material
