# Supplementary material for: Comparison of the performance of a clinical classification tree versus clinical gestalt in predicting sepsis with extended-spectrum beta-lactamase–producing gram-negative rods
Source: Antimicrob Steward Healthc Epidemiol. 2022 Mar 7;2(1):e35. doi: 10.1017/ash.2021.253 (PMC9614789; doi:10.1017/ash.2021.253)

**Supplemental Document 1:**

**Extended spectrum beta-lactamase testing process**

Extended spectrum beta-lactamase (ESBL) is tested for in E. coli, Klebsiella pneumoniae, Klebsiella oxytoca, and Proteus mirabilis using phenotypic methods (i.e. elevated minimum inhibitory concentrations [MICs]).

For K. pneumoniae, K. oxytoca and E. coli the laboratory screened for the following: cefpodoxime of > 4ug/ml, or ceftazidime > 1 ug/ml, or aztreonam >1 ug/ml, or cefotaxime >1 ug/ml or ceftriaxone > 1 ug/ml. For P. mirabilis, looked for MIC's: cefpodoxime >1 ug/ml, ceftazidime > 1 ug/ml, or cefotaxime > 1 ug/ml. If any of these were elevated, it indicated the possible presence of an ESBL. For confirmation, the laboratory performed MIC testing using ceftazidime concentrations of 0.24- 128 ug/ml and ceftazidime-clavulanate concentrations of 0.25/4-64/4. The laboratory also tested cefotaxime concentrations of 0.25-64 ug/ml and cefotaxime-clavulanate concentrations of 0.25/4- 64/4 ug/ml. They used broth microdilutions in doubling dilutions.

A >=3 twofold concentration decrease in MIC for either antimicrobial agent testing in combination with clavulanate versus the MIC of the agent when tested alone indicated the presence of an ESBL (e.g., ceftazidime MIC = 8 ug/ml; ceftazidime-clavulanate MIC = 1 ug/ml).

For Enterobacter spp, Citrobacter spp, Morganella morganni and Serratia marcescens we used resistance to ceftriaxone as a marker for presence of ESBL

**Supplemental Document 2:**

Modeling threats related to imbalanced outcomes can result in an imbalance between false negative and false positive error rates. To control for this concern, trees were fit using a cost matrix, which assigned false negatives a penalty twelve times larger than false positives. Gini Index values were used as the criteria for tree splits and pruning based on 10-fold cross-validation error rates for the selection of a regularized tree fit based on cost complexity values.

The final pruned tree was fit to the full dataset and ESBL-PE screening metrics included: sensitivity, specificity, positive predictive value (PPV), negative predictive value (NPV), positive likelihood ratio (PLR), negative likelihood ratio (NLR), and accuracy. These values along with a calibration curve were presented with 95% confidence intervals (CI). When reporting rate differences for the ESBL-PE tree, a Bonferroni correction was applied to the 0.05 alpha level. The area under the receiver operating characteristic curve (AUROCC) for the final tree was contrasted with both the AUROCC for patients prescribed a carbapenem antibiotic by the ED or admitting hospital-based clinician. The former comparison was also presented as a Shannon information binary surprise index (*S*-value), and Brier scores were also calculated for these two trees. A planned sensitivity analysis was conducted via the replication of the above tree building process using balanced prior probabilities (50:50) for ESBL-PE status in lieu of a cost-matrix.

**R syntax for naïve tree (not published), employed tree, and sensitivity analysis tree**

library(rpart); library(rpart.plot); library(rattle)

#############################################################

# 1.) First Tree: Not addressing dependent variable imbalance

# Naive (Crude) Tree – Not used in paper.

#############################################################

set.seed(2019)

Crude_Tree <- rpart(esbl ~ age + cvc_present + long_term + ABX + ho_esbl,

data = esbl_paperer, method = "class", minsplit = 2)

plot(Crude_Tree, uniform=TRUE, main="Classification Tree for ESBL")

text(Crude_Tree, use.n=TRUE, all=TRUE, cex=.8)

Crude_Tree

summary(Crude_Tree)

Crude_Tree$variable.importance

# Initial Tree (Pruning)

# Using cp to trim: cp = complexity parameter, based on 10-fold cross-validated

printcp(Crude_Tree)

plotcp(Crude_Tree)

set.seed(2019)

Crude_Tree_Pruned <- prune(Crude_Tree, cp= Crude_Tree$cptable[which.min(Crude_Tree$cptable[,"xerror"]),"CP"])

plot(Crude_Tree_Pruned, uniform=TRUE, main="Classification Tree for ESBL")

text(Crude_Tree_Pruned, use.n=TRUE, all=TRUE, cex=.8)

fancyRpartPlot(Crude_Tree_Pruned)

Crude_Tree_Pruned$variable.importance

##############################################################################

# 2.) Second Tree: Addressing DV imbalance using cost matrix

# False negatives 12X worse than false positives

##########################################################################

loss <- matrix(c(0, 12, 1, 0), ncol=2)

set.seed(2019)

Loss_Matrix_Tree <- rpart(esbl ~ age + cvc_present + long_term + ABX + ho_esbl,

data = esbl_paperer, method = "class", minsplit = 2, parms=list(loss=loss))

plot(Loss_Matrix_Tree, uniform=TRUE, main="Classification Tree for ESBL")

text(Loss_Matrix_Tree, use.n=TRUE, all=TRUE, cex=.8)

Loss_Matrix_Tree

summary(Loss_Matrix_Tree)

Loss_Matrix_Tree$variable.importance

printcp(Loss_Matrix_Tree)

plotcp(Loss_Matrix_Tree)

Loss_Matrix_Tree_Pruned = prune(Loss_Matrix_Tree, cp=Loss_Matrix_Tree$cptable[which.min(Loss_Matrix_Tree$cptable[,"xerror"]), "CP"])

plot(Loss_Matrix_Tree_Pruned, uniform=TRUE, main = "Pruned Tree - Entropy")

text(Loss_Matrix_Tree_Pruned, use.n=TRUE, all=TRUE, cex=0.8)

rpart.plot(Loss_Matrix_Tree_Pruned)

Loss_Matrix_Tree_Pruned$variable.importance

#############################################################################

# 3.) Third Tree: Addressing DV imbalance using prior weights

# Uses Priors (cprior): weight scheme, default is empirical outcome values

#############################################################################

set.seed(2019)

summary(esbl_paperer$esbl)

cprior_Tree <- rpart(esbl ~ age + cvc_present + long_term + ABX + ho_esbl,

data = esbl_paperer,

method = "class",

minbucket = round(5/2),

parms=list(prior=c(0.5, 0.5)))

plot(cprior_Tree, uniform=TRUE, main="Classification Tree for ESBL")

text(cprior_Tree, use.n=TRUE, all=TRUE, cex=.8)

cprior_Tree

summary(cprior_Tree)

cprior_Tree$variable.importance

# cprior Tree (Pruning)

printcp(cprior_Tree)

plotcp(cprior_Tree)

set.seed(2019)

cprior_Tree_Pruned <- prune(cprior_Tree, cp= cprior_Tree$cptable[which.min(cprior_Tree$cptable[,"xerror"]),"CP"])

plot(cprior_Tree_Pruned, uniform=TRUE,

main="Classification Tree for ESBL")

text(cprior_Tree_Pruned, use.n=TRUE, all=TRUE, cex=.8)

fancyRpartPlot(cprior_Tree_Pruned)

cprior_Tree_Pruned$variable.importance

**Supplemental Figure 1.** Age of patients suspected of bacteremia receiving a blood culture in the investigation of extended spectrum beta-lactamase (ESBL) infection. Of these patients, 565 (91%) were not positive for ESBL (top pane) and 56 (9%) were ESBL positive (bottom pane).


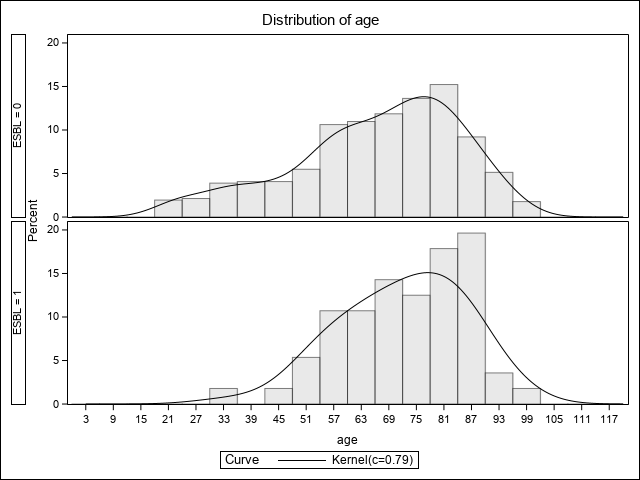


**Supplemental Figure 2.** Alternative visualization of the classification tree fit based on Emergency Department point of care information available in patients suspected of bacteremia and received blood culture from a study investigating extended spectrum beta-lactamase (ESBL) Infection, N=621. Definitions: ho_esbl = documented history of ESBL in prior 2-years and long_term = healthcare residence.


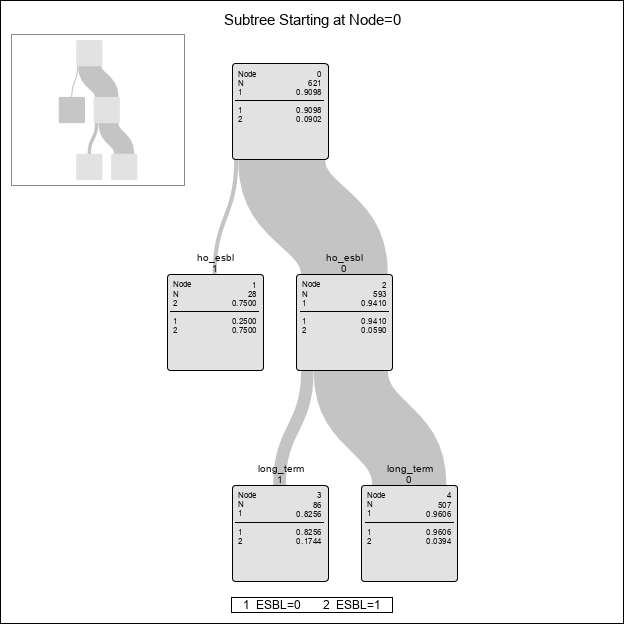


**Supplemental Figure 3.** Receiver operating characteristic curve for patients suspected of bacteremia receiving blood culture in a study investigating extended spectrum beta-lactamase (ESBL) Infection (N=621). Fitted are ESBL classification based on carbapenem received in the Emergency Department (ED), empiric carbapenem received by admitting service, and the application of a point of care-based classification tree. The Brier Scores for the constructed tree and whether the patient received a carbapenem through the ED were 0.06 and 0.08, respectively.


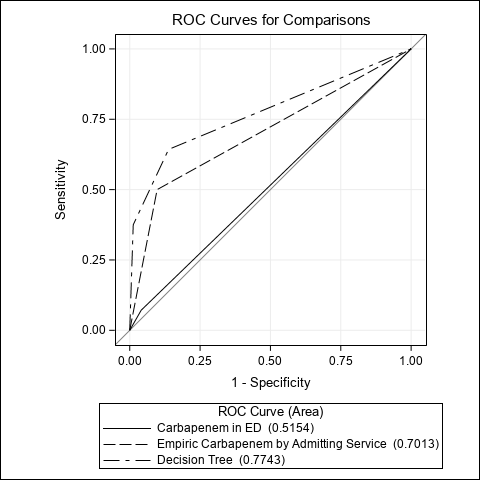


**Supplemental Figure 4.** Calibration curve based on applying the classification tree to the full dataset, presented with 95% confidence intervals. Y-axis represents percentage of patients with positive status for extended spectrum beta-lactamase (ESBL) versus X-axis representing predicted probability given tree splits based on Emergency Department patients suspected of bacteremia having a blood culture collected, N=621.


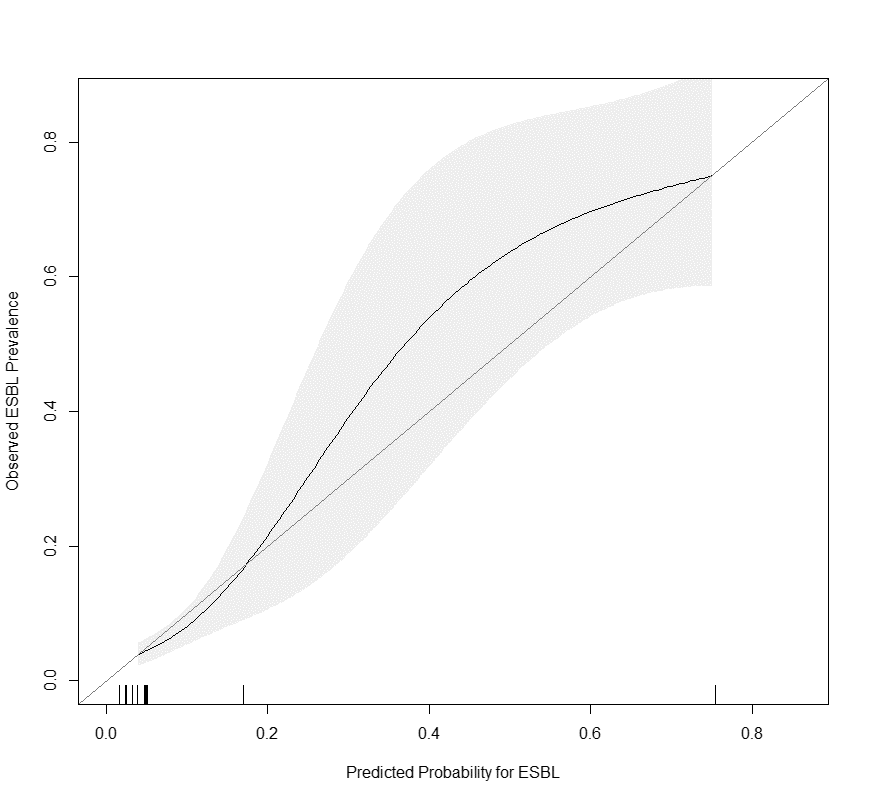

Supplement: Supplementary file 1 [file S2732494X21002539sup001.docx]
